# Meeting of the DeWitt County Medical Society

**Published:** 1859-05

**Authors:** John McHugh

**Affiliations:** Secretary


### MEETING OF THE DE WITT COUNTY MEDICAL SOCIETY.

The Society met in annual session at the office of Dr. Goodbrake, in Clinton, on Monday, the 4th day of April, 1859, at 10 o'clock A. M. Present, Drs. John Wright, T. K. Edmiston, John McHugh, W. W. Adams, B. S. Lewis, John Tyler, Z. H. Madden, C. Goodbrake, H. Noble; also, Dr. R. G. McLaughlin, of Heyworth. Dr. Wright, President, in the chair. The Secretary being absent, Dr. Goodbrake was appointed Secretary pro tem.

The minutes of the last meeting were read and approved.

On motion of Dr. Goodbrake, Dr. R. G. McLaughlin was elected an Honorary Member of this Society. The Doctor thanked the Society in a very handsome manner for the honor conferred.

The following gentlemen were elected officers of the Society for the ensuing year:

*President*, . . . Dr. B. Lewis,
*Vice-President*, . . Dr. D. K. Edmiston.
*Treasurer*, . . . Dr. John H. Tyler.
*Secretary*, . . . Dr. John McHugh.

*Censors*, Drs. C. Goodbrake, John Wright, and Mr. W. Adams.

Drs. John McHugh and J. H. Tyler were elected delegates to the American Medical Association.

Drs. John Wright, C. Goodbrake, W. W. Adams and J. K. Edmiston were elected delegates to the Illinois State Medical Society.

Dr. Wright, the retiring President, delivered a very excellent valedictory address, on the subject of tobacco; which was well received, and the subject discussed by most of the members present; and the Secretary ordered to furnish a copy of said address to the *Chicago Medical Journal*, for publication.

John A. Edmiston, medical student, read an essay on the action of medicines, which was ordered on file.

On motion, the Secretary was ordered to furnish a copy of the proceedings of this meeting to the editors of the *Chicago Medical Journal*, with the request to publish the same.

On motion, the Society adjourned, to meet in quarterly session, at Waynesville, on the first Monday in July next, at ten o'clock A.M.

JOHN McHUGH, M.D., *Secretary*.

